# Prospective Assessment of Early Proton Therapy-Induced Optic Neuropathy in Patients With Intracranial, Orbital or Sinonasal Tumors: Impact of A Standardized Ophthalmological Follow Up

**DOI:** 10.3389/fonc.2021.673886

**Published:** 2021-06-15

**Authors:** Marie Lecornu, Paul Lesueur, Julia Salleron, Jacques Balosso, Dinu Stefan, William Kao, Tiphaine Plouhinec, Anthony Vela, Pauline Dutheil, Jordan Bouter, Pierre-Alban Marty, Juliette Thariat, Jean-Claude Quintyn

**Affiliations:** ^1^ Radiation Oncology Department, Centre François Baclesse, Caen, France; ^2^ Radiation Oncology Department, Centre Guillaume le Conquérant, Caen, France; ^3^ ISTCT UMR6030-CNRS, CEA, Université de Caen-Normandie, Equipe CERVOxy, Caen, France; ^4^ Cellule Data Biostatistique, Instistut de Cancerologie de Lorraine, Nancy, France; ^5^ Radiation Oncology Department, Institut Universitaire du Cancer de Toulouse, Toulouse, France; ^6^ Ophthalmology Department, University Hospital of Caen, Caen, France

**Keywords:** proton therapy, radiation-induced optic neuropathy, optic toxicity, optic pathway tolerance, radiation neuropathy

## Abstract

**Purpose:**

Proton therapy (PT) can be a good option to achieve tumor control while reducing the probability of radiation induced toxicities compared to X-ray-based radiotherapy. However, there are still uncertainties about the effects of PT on the organs in direct contact with the irradiated volume. The aim of this prospective series was to report 6-month follow-up of clinical and functional optic neuropathy rates of patients treated by proton therapy using a standardized comprehensive optic examination.

**Methods and Materials:**

Standardized ophthalmological examinations were performed to analyze subclinical anomalies in a systematic way before treatment and 6 months after the end of proton therapy with: Automatic visual field, Visual evoked potential (VEP) and optic coherence of tomography (OCT).

**Results:**

From October 2018 to July 2020 we analyzed 81 eyes. No significant differences were found in the analysis of the clinical examination of visual functions by the radiation oncologist. However, considering VEP, the impairment was statistically significant for both fibers explored at 30’angle (p:0.007) and 60’angle (p <0.001). In patients with toxicity, the distance of the target volume from the optical pathways was more important with a p-value for 30’VEP at 0.035 and for 60’VEP at 0.039.

**Conclusions:**

These results confirm uncertainties concerning relative biological effectiveness of proton therapy, linear energy transfer appears to be more inhomogeneous especially in areas close to the target volumes. The follow-up of patients after proton therapy is not an easy process to set up but it is necessary to improve our knowledges about the biological effects of proton therapy in real life. Our study which will continue during the coming years, suggests that follow-up with in-depth examinations such as VEP as a biomarker could improve the detection of early abnormalities.

## Introduction

The treatment of skull base tumors often relies on surgery and radiotherapy (1, 2). The delivery of a high dose to the tumor nearby organs at risk (OAR) such as optic nerves and chiasma can be challenging. Consequences of radiation induced optic toxicities are various and may induce a loss of visual acuity, visual field disorder or retinopathy. These anomalies can appear from 3 months to 10 years after radiotherapy (3). Radiation-induced optic neuropathy (RION) is defined by a painless defect of visual acuity, in one or both eyes after a latency of months to years after radiotherapy (4). Due to damages to the optic nerve, the visual field is reduced to a variable extend. According to the literature, optical toxicities appear from a cumulative dose between 55 to 60Gy (EQD2) or for single fraction greater than 10Gy (5–7). Most treatments are still carried out by X-ray. Although new photonic techniques allow excellent coverage of target volume and a better respect of OAR’s dose constraints (8–10) than conventional technics. In complex or large tumors involving areas in direct proximity of the anterior optic pathways, damage to the optic structures may be frequent and somehow unavoidable. The advantageous dose distribution of proton may be used to spare OARs located close to the tumor (11–13) and to spare healthy brain. In such cases, the aim of proton therapy (PT) is to achieve tumor control while reducing the probability of radiation induced toxicities compared to modern X-ray-based radiotherapy (RT), such as intensity modulated radiation therapy (IMRT) or stereotactic radiation therapy (SRT). However, there are still uncertainties about the effects of PT on the organs in direct contact with the irradiated volume, in particular regarding relative biological effectiveness (RBE). Indeed RBE could be underestimated at the beam end due to high linear energy transfer (LET) (14). Proton therapy series report a 7% risk to develop severe optic neuropathy (15). These rates may be underestimated because data are generally based on patient’s spontaneous reporting instead of systematic and standardized collection. Moreover, these optic toxicities do not appear to follow the previously established dose volume effects for optic neuropathy, based on photons irradiation (X-rays). Consequently, proton induced optic toxicities may differ from those of photons. Only few data are published, mainly retrospective (15–18). As an attempt to fill this gap we designed, in the context of our rising proton therapy activity, a prospective and comprehensive assessment of optic outcomes using a standardized optic workup at baseline and during patients’ follow up. The aim of this study was to find predictive early subclinical alterations because when radiation induced optic disorder are clinically significant, they are unfortunately irreversible. An additional aim, at the populational scale could be to contribute to improve biomathematical modelling for outcome predictions, and therefore treatment planning optimization, for safer treatments.

Accordingly, this prospective series is an initial and preliminary report of 6-month follow-up of clinical and functional optic neuropathy rates of patients treated by proton therapy using a standardized comprehensive optic examination.

## Methods and Materials

### Population Analysis

The first comprehensive optic examination (optic Work-up) was performed in October 2018. Patients ≥ 18 years old, with tumors (benign or malignant) within 1cm of the optic tract were included in this Institutional Review Board approved study, after multidisciplinary staff meeting and technical expert committee meeting. An information letter is sent to each patient informing them that data from patients treated with proton therapy in Caen were related to clinical research. Patients were referred from multiple institutions but all were treated at the Normandy proton therapy center (Caen, France) and underwent optic examinations at the University hospital of Caen. Exclusion criteria were pediatrics, secondary or intraocular tumors and refusal to undergo optic examinations. To properly assess dose volume effects and the impact of previous damage from tumor, surgery, or any other treatment on the optic nerves, chiasm, and other optic structures, we adjusted outcomes on status before PT. Past medical history, treatments and comorbidities were reported, some have been recognized as risk factors for RT induced toxicities: diabetes, hypertension, glaucoma, smoking for example.

### Tumors and Location

Tumor diagnoses were distributed into different groups: meningiomas, pituitary adenoma, craniopharyngioma and other rarer diagnoses. The minimal distance to optical structures was assessed and tumors were separated in three groups: those invading or abutting the optic pathways, tumors located between 0 and 3mm from the optic pathway and tumors between >3 and 10mm.

### Treatment

Tumor and OAR delineation was based on millimetric CT scan and multimodal imaging including systematically a contrast enhanced fusion MRI in treatment position. Proton therapy was performed using pencil beam scanning (PBS) with a ProteusOne^®^ machine (IBA, Louvain la Neuve, Belgium). PBS corresponds to a three dimensions scanning obtained by successive plan scanning done by individually modulated mono-energy beams of adapted energy (19). First treatments were delivered in IMPT (Intensity Modulated Proton therapy) with Single field optimization (SFO) and after one year, IMPT multiple field optimization (MFO) was applied when necessary, depending on OAR (Organ at risk) constraints (20). During the planning process we checked that the end of the beam range was never in front of the optical structures. The intensity of the spots and their location were analyzed. In case of positioning of spots too intense in the heart of a volume at risk this was modified. The LET mapping was not routinely performed before treatment because the software did not allow it. The treatment plan was calculated using robust optimization (assuming 3mm positioning uncertainty and 3% proton range uncertainty unless filling cavity uncertainties) (21) using the Treatment planning system (TPS) Raystation (Raysearch^®^). We performed also a robust evaluation. Calculations includes a 1.1 relative biological effectiveness. Treatment was delivered in 1.8-2Gy (RBE) fractions, five days a week. Some patients benefited from a combination of photon and proton treatment. In some cases, a boost dose of 12 to 21.6Gy in 2.4Gy per fraction was delivered by SRT by a Cyberknife^®^ machine (Accuray, Madison, WI, USA). These were applied because they represented a dosimetric advantage. In the event of beam downtime and so as to hold the tumor control probability, some patients underwent photon-based replanning until PT resumed.

### Practical Ophthalmological Examinations

A standard clinical examination was carried out by radiation oncologist including: subjective deficit of visual acuity, oculomotor nerve disorders and visual field disorder. Then, he prescribed the complementary follow-up examinations. Each patient was addressed to the ophthalmologist who performed in first, clinical exam with: photo-motor reflex, a measure of visual acuity, a dilated fundus and a measure of lens opacity.

More specific examinations were systematically performed: a visual field exam, papillary optical coherence tomography (OCT), and visual evoked potentials.

The visual field corresponds to the entire area that a person can see when looking at a point. The average corrected deficit was collected allowing to make a discrimination between normal and pathological examinations and specially to obtain a follow-up for each patient. If the average corrected deficit was more than 3 points different from the general population, the result was considered pathological. Visual field tests results can help to determine the location of the radiation-induced damage. In order to specify the available data concerning the visual field, the perimetry has been detailed in 6 sectors to allow locating the anomalies: nasal, upper nasal, lower nasal, temporal, lower temporal and upper temporal.

**Figure 1:**
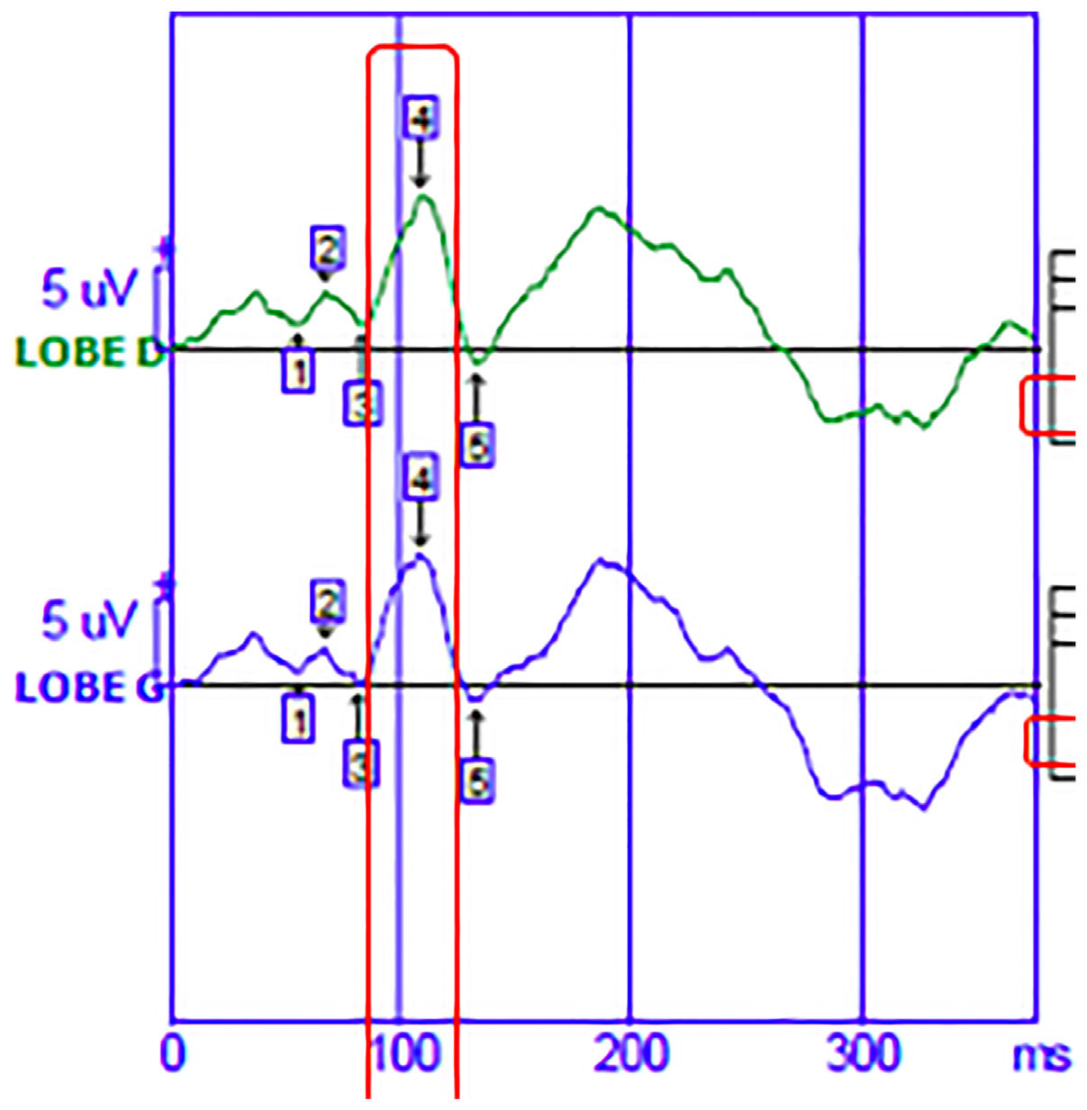
Visual evoked potential.The green curve corresponds to the nerve signal born in the visual cortex of the right occipital lobe. The blue curve corresponds to the nerve signal born in the visual cortex of the left occipital lobe.The abscissa shows the time in milliseconds. On the ordinate is the amplitude of the wave in microvolt.

Patients also benefit from a measurement of visual evoked potentials (VEP). VEP is the physiological response of the occipital cortex to a sensory stimulus of vision. Latency and amplitude are evaluated. VEP provide information about optic neuritis (22). We collected P100 data for each eye and for the 60’ and 30’ visual angles to obtain data from different macular fibers. The latency of the P100 (**Figure 1**) wave was considered pathological if it was greater than 120ms. The amplitude was considered pathological if it was less than 6 microvolts. If the disorders were symmetrical on the right and left occipital lobe, it was an impairment of the anterior optic pathways.

The Optical coherence tomography (OCT) was performed to obtain high resolution images of the retina. Measurement of retinal nerve fiber layers (RNFL) is used to assess optic nerve fiber damage. A fiber thickness of less than 60µm was considered pathological.

At the end of this paraclinical assessment, the patient was seen in consultation with an ophthalmologist.

### Follow-Up

Prospective assessment was performed at baseline, i.e. before PT, 6 and 12 months after the end of treatment, and every year. Clinical exam and MRI or CT were performed every 3 months after PT.

### Statistical Analysis

Qualitative parameters were described as frequency and percentage, quantitative parameters as median and interquartile range. Normality of the distribution was investigated with Shapiro-Wilks test. The comparisons of qualitative parameters from T0 to T6 were performed with Mac-Nemar test (paired Chi-squared test). Eyes with and without toxicity were compared by Chi-squared test or Fisher Exact test for qualitative parameters and with Wilcoxon U test for quantitative ones.

Significance level was set at 0.05. Statistical analyses were performed with SAS software, version 9.4 (SAS Institute Inc., Cary, NC, USA.).

## Results

From October 2018 to July 2020 we recruited 41 patients for this study. Sixty patients were initially eligible, but 2 patients were deceased, 2 were too impaired to perform the follow-up. One patient was no longer living in France, 7 ophthalmological examinations has been cancelled because of the global pandemic COVID-19 and 7 exams are missing because the patients did not show up. The median follow-up time was 8 months, interquartile range from 7 to 9 months. In **Table 1** are summarized the patients’ baseline characteristics. Most patients were treated for meningiomas (53.7%). Clinical deficits were initially in 13 (31.7%) patients. The median age was 57 years old {19-92}. Most of patients (73.2%) had already undergone at least one local treatment: 27 patients (65.9%) were treated with at least one surgery and 3 (7.3%) with previous radiotherapy. We included these 3 patients because the doses received at the previous optical structures were not significant. Two patients had a rather distant irradiation (maxillary and cervical). The third patient had already received 66Gy on the same volum but had no toxicity from his previous irradiation 5 years earlier. The eleven remaining patients received radiotherapy as first line treatment. The tumor abutted or invaded the optic tract (in the case of malignant tumors) in 23 patients (56%), i.e., the distance to the optic tract was 0mm on MRI. Considering PT, the majority of patients received single field SFO (single field optimization) type PT (92.7%). Some patients received treatment with photon. On 41 patients, 18 patients received a combined photon-proton therapy. For 15 patients it was due to machine failure. Three patients had benefited from an additional dose of 12 and 21.6Gy in stereotactic condition with 2.4Gy per fraction. Boost were applied because it was represented a dosimetric advantageous. The **Table 2** summarized the treatment characteristics of the patients.

**Table 1:** Population baseline characteristics.

	n (%)
**WHO performance status**	
0	30 (73.2)
1	9 (21.9)
2	2 (4.9)
**Age (years),** Median [interquartile range]	57 [43; 63]
**Sex**	
Male	15 (36.6)
Female	26 (63.4)
**Months since diagnosis:** Median [interquartile range]	12[4-50]
**Comorbidities**	22 (53.7)
Diabetes	3 (7.4)
Hypertension	10 (24.4)
Smoking	10 (24.4)
Vascular disease	6 (14.6)
**Histology**	
Meningioma	22 (53.7)
Adenoma/craniopharyngioma	7 (17.1)
Other	12 (29.3)
**Initial deficits**	13 (31.7)
**Previous treatment history**	28 (68.3)
Radiotherapy	3 (7.3)
Surgery	27 (65.9)
**Enucleation**	1 (2.4)
**Medical treatments**	4 (9.8)
Chemotherapy	3 (7.5)
Immunosuppressor	1 (2.4)
**Distance of optical structures**	
0mm	23 (56)
0-3mm	8 (19.5)
>3mm	10 (24.4)
**Glaucoma**	2 (4.9)
**Clinical exam**	
Neurological deficit	17 (41.5)
Oculomotor deficit	12 (29.3)
VA deficit	10 (24.4)
VF deficit	9 (22)

WHO, world health organization performance status; n, number of patients; VA, visual acuity; VF, visual field.

**Table 2:** Characteristics of treatment for 41 patients.

	n (%)	Median (range)
**Proton only**	23 (56)	
**Proton + photon**	18 (44)	
**SFUD**	36 (87.8)	
**IMPT**	5 (12.2)	
**Number of beams**		
**1**	1 (2.4)	
**2**	38 (92.7)	
**3**	2 (4.9)	
**Volume CTV (cm3)**		26.7 (6.8 to 237.4)
**Prescription dose (Gy (RBE))**		54 (24 to 73.8)

n, number of patients; SFUD, single field uniform dose; IMPT, intensity modulated proton therapy; CTV, clinical target volume; RBE, relative biological efficiency (considered as 1.1 for protons).

### Radiation Oncologist Examination

We analyzed specifically the evolution of results of the clinical examination by the radiation oncologist. No significant difference was found. Results clinical examination by radiation oncologist at baseline and after 6 months were summarized in **Table 3**.

**Table 3:** Clinical examination at 0 and 6 months.

	Baseline	At 6 months	p-value
Oculomotor deficit	12	11	0.66
VA deficit	10	4	0.083
VF deficit	9	6	0.37

VA, visual acuity; VF, visual field.

### Ophthalmological Examinations

Results of ophthalmological examinations are summarized in **Table 4**. They were analyzed by eye, so we had 81 eyes for 41 patients because 1 patient undergone an enucleation. Concerning baseline’s results, 11% of patients had a loss of visual acuity. At 6 months, there was no significant difference since visual deficit was found in 12.4% of eyes. Concerning the visual field, the impairment severity decreased over time. In fact, the number of eyes with more than 3 sectors affected was 8.6% at 6 months compared to 23.5% at baseline. However, the analysis of the mean corrected deficit did not show a significant difference with p-value: 0.317.

**Table 4:** Summary of abnormal result of each ophthalmological exam for 81 eyes.

DEFICIT	T0	T6	p-value
**VA**	9 (11.1%)	10 (12.35%)	0.564
**VF**	23 (30.3%)	16 (21.9%)	0.317
**Number of sectors impaired:**			
0	37 (45.68%)	50 (61.73%)	0.027
1-2	15 (18.52%)	15 (18.52%)	
3	10 (12.35%)	9 (11.11%)	
>3	19 (23.46%)	7 (8.64%)	
**Cataract**	27 (33.33%)	31 (38.3%)	0.165
**30’ VEP**	33 (41.8%)	47 (59.5%)	0.007
**60’ VEP**	27 (33.3%)	44 (55.7%)	<0.001
**OCT**	12 (15.6%)	11 (14.9)	1

T0, number of deficits at baseline; T6, number of deficits at 6 months; VA, visual acuity (abnormal if ETDRS <55); VF, visual field (pathological if average corrected deficit different by 3 points from general population); OCT, optical coherence tomography (abnormal if thickness < 60 micrometers); VEP, visual evoked potentials (abnormal if amplitude < 6 µV or latency > 120ms).

The initial deficit of VEP for 30’ fibers represented 33 eyes (41.8%) and at 6 months VEP deficit affected 47 eyes (59.5%). Twenty-seven (33.3%) 60’ VEPs were abnormal at baseline and 44 (55.7%) at 6 months. This impairment was statistically significant for both fibers explored at 30’ and 60’ angles with a p-value of 0.007 and <0.001 respectively.

Analysis of the results for optical coherence tomography (OCT) did not reveal any significant difference.

On Fifty-three 60’ VEP normal at baseline 21 became pathological after 6 months of follow-up. For the 30’ VEP, 45 eyes were normal prior to treatment, and 18 of them developed toxicity. We compared for the 30’ VEP and 60’ VEP those that remained normal compared to those that became pathological after proton therapy.

Distance from optical structures was a significant factor influencing the evolution of VEP in both groups, with a p-value for 30’VEP at 0.035 and for 60’VEP at 0.039. In fact, patients whose tumors were in direct contact with the optical pathways, 30’ VEP and 60’ VEP were less affected.

Treatment history i.e. radiotherapy or surgery were more frequent for patients with toxicity on 30’ VEP (p= 0.017). The results are summarized in **Table 5**.

**Table 5:** Comparison of patients who experienced VEP toxicity to those who remained normal, analyses for VEP 30’.

	Eyes with toxicity n=18	Eyes without toxicity n=27	p-value
**Age (years): Median [interquartile range]**	47.4 [41.7;60.5]	58.4 [50.4;60.8]	0.224
**Time from diagnostic**	46[11;61]	14 [2;51]	0.168
**Time from baseline**	8.8 [7;9.4]	7.8 [5.8;8.9]	0.242
**Sex**			
1	8 (44.4%)	6 (22.2%)	0.115
2	10 (55.6%)	21 (77.8%)	
**Comorbidities**	9 (50.0%)	17 (63.0%)	0.388
**Histology**			0.140
Meningioma	9 (50.0%)	21 (77.8%)	
Adenoma/craniopharyngioma	4 (22.2%)	2 (7.4%)	
Other	5 (27.8%)	4 (14.8%)	
**Initial deficits**	8 (44.4%)	10 (37.0%)	0.620
**Treatments history**	15 (83.3%)	13 (48.1%)	0.017
**Distance of optical structures**			0.035
0mm	5 (27.8%)	18 (66.7%)	
0-3mm	5 (27.8%)	4 (14.8%)	
>3mm	8 (44.4%)	5 (18.5%)	
**Clinical exam**			
Neurological deficit	6 (33.3%)	13 (48.1%)	0.324
Oculomotor deficit	5 (27.8%)	12 (44.4%)	0.259
VA deficit	2 (11.1%)	5 (18.5%)	0.502
VF	3 (16.7%)	3 (11.1%)	0.670
Other	2 (11.1%)	1 (3.7%)	–
**CTV (cm3)**	35.7 [10.9;73.2]	24.3 [12.6;53.1]	0.108
**Prescription dose**	54 [54;59.4]	54 [54;54]	0.447
**D1% chiasma**	51.3 [47.7;52.1]	51.7 [39.2;52.4]	0.651
**D2% chiasma**	51.1 [47.2;52]	51.6 [38.9;52.2]	0.577
**D50% chiasma**	48.2 [40.6;50.7]	44.3 [29.7;50.9]	0.685
**Dose per fraction chiasma**	1.7 [1.5;1.7]	1.7 [1.2;1.8]	0.468
**D1% ON**	51.1 [33.5;52.1]	51.6 [11.9;52.2]	0.790
**D2% ON**	51 [31.5;52]	51.1 [9.6;52.1]	0.790
**D50% ON**	18.4 [1.4;39]	13.5 [0.4;38.8]	0.635
**Dose per fraction ON**	1.6 [1.1;1.7]	1.7 [0.4;1.7]	0.785

D1%, D2%, D50% are respectively the doses received by 1%, 2% or 50% of an irradiated volume. ON, Optic nerve.

For 60’ VEP, the dose received by 50% of the volume of the chiasma (D50%) was significantly higher in patients with toxicity (p=0.04), the D2% for optic nerve was also more important, 51.2Gy for eyes with toxicity and 41.9Gy for eyes free of injury, p: 0.042. Age appeared to be an influenced factor with p= 0.057. The results are summarized in **Table 6**.

**Table 6:** Comparison of patients who experienced VEP toxicity to those who remained normal, analyses for VEP 60’.

	Eyes with toxicity n=21	Eyes without toxicity n=32	p-value
**Age (years): Median [interquartile range]**	60.5 [42.8;75]	52.8 [37;60.2]	0.057
**Time from diagnostic**	14.2 [4.2;49.3]	14 [2.3;54.7]	0.723
**Time from baseline**	8.9 [7.6;9.2]	8.5 [6.8;9.5]	0.461
**Sex**			
1	9 (42.9%)	12 (37.5%)	0.696
2	12 (57.1%)	20 (62.5%)	
**Comorbidities**	10 (47.6%)	16 (50.0%)	0.865
**Histology**			0.757
Meningioma	10 (47.6%)	18 (56.2%)	
Adenoma/craniopharyngioma	4 (19.1%)	4 (12.5%)	
Other	7 (33.3%)	10 (31.3%)	
**Initial deficits**	8 (38.1%)	11 (34.4%)	0.782
**Treatments history**	15 (71.4%)	22 (68.7%)	0.835
**Distance of optical structures**			
0mm	6 (28.6%)	16 (50.0%)	0.039
0-3mm	9 (42.9%)	4 (12.5%)	
>3mm	6 (28.6%)	12 (37.5%)	
**Clinical exam**			
Neurological deficit	8 (38.1%)	12 (37.5%)	1
Oculomotor deficit	8 (38.1%)	10 (31.2%)	0.607
VA deficit	3 (14.3%)	4 (12.5%)	1
VF deficit	3 (14.3%)	3 (9.4%)	–
Other	1 (4.8%)	2 (6.2%)	–
**CTV (cm3)**	34 [12.6;42.4]	25.5 [11.8;107.6]	0.518
**Prescription dose**	54 [54;54]	54 [54;56.7]	0.992
**D1% chiasma**	51.1 [49.3;52.3]	50.6 [14;52.3]	0.164
**D2% chiasma**	50.9 [49.3;52.2]	50.1 [12.3;52.1]	0.148
**D50% chiasma**	48.2 [40.6;50.9]	42 [1.8;49.2]	0.040
**Dose per fraction chiasma**	1.6 [1.5;1.7]	1.6 [0.4;1.7]	0.494
**D1% ON**	52 [43.4;52.3]	43.3 [15.4;52]	0.028
**D2% ON**	51.2 [43;52.2]	41.9 [13.6;51.8]	0.042
**D50% ON**	22.9 [10.8;36.5]	8 [0.4;33.1]	0.120
**Dose per fraction ON**	1.6 [1.4;1.7]	1.5 [0.4;1.7]	0.098

D1%, D2%, D50% are respectively the doses received by 1%, 2% or 50% of an irradiated volume. ON, Optic nerve.

## Discussion

Optical toxicities may limit the treatment of tumors of the skull-base. Proton therapy is a good option to reduce the irradiated volume of healthy brain. However, there are still some debate about the effects of proton therapy on the organs in direct contact with the irradiated volume, particularly because of uncertainties of RBE and linear energic transfer. In the treatment of para optic tumors, RION is the main limiting toxicity and its detection needs a careful follow-up so as to ensure an early detection and ophthalmological care. Unfortunately, data for monitoring visual function after these treatments are rare and incomplete (23). The difficulty of follow-up is mainly related to the limited number of proton therapy centers, so the treated population comes from different remote regions and patients lost to follow-up are frequent. We tried to obtain a large amount of data by doing the follow-up in a systematic way but in nearly 30% of cases data of 6 months examinations were lacking. It can be explained by the asymptomatic nature of impairment, patients are less motivated for follow up. Eight ophthalmological examinations have been cancelled because of the global pandemic COVID-19. Specific ophthalmological examinations were performed to analyze subclinical anomalies. The results presented are exploratory.

As shown by our results, the clinical examination of the radiation oncologist is not a sensitive examination for the detection of abnormalities or improvements in visual functions.

Overall, with 6 months of delay we did not observe any significant increase in optical damage after proton therapy, this suggests that proton therapy might be a safe and well-tolerated treatment, at least at short term. This is in agreement with the results of previous studies that have demonstrated the safety of proton therapy on visual function at early follow-up.

To our knowledge, few studies have been conducted to analyze the subclinical effects and long-term consequences of proton therapy on visual functions. Moreover, these studies were based on retrospective data and the ophthalmological examinations were performed only in case of clinical abnormality in a non-standardized way. In 2018, Li *and al* published a study evaluating visual functions after PT for chordoma and chondrosarcoma, the results involved a large number of patients with a long-term follow-up of 4.8 years. Considering retrospective and not standardized data, the low level of optical toxicity reported was probably underestimated: only 1% among patients receiving <59Gy (RBE) and 5.8% among patients receiving ≥ 60Gy (RBE) to the optic pathway developed optic toxicities. In this study, RION was defined as the loss of visual acuity. However in optic neuropathy, the decrease of visual acuity is a late sign that is not necessary for early diagnosis (24).

El Shafie et al. (25), in a prospective study, suggested that proton therapy was an effective and safe treatment, but only acute toxicities were assessed. In Kountouri’s data published in 2019, optic toxicity was rare with 7% of patients developing optic troubles (15), but most of them were severe (8 patients of 14 with grade IV RION). Given that, in this study, patients did not benefit from a full standardized ophthalmologic follow up, it suggests that much more patients could present infra clinical or low-grade optic impairment.

In our study, the assessment of visual fields showed an improvement between baseline and the 6-month examination. These results could be biased by the patient’s learning curve and requires more follow-up to characterize the objective evolution over time. The validity of information obtained from a visual field test depends on the ability of the patient to perform the test correctly with caution (26). According to studies carried out by ophthalmologists as part of glaucoma follow-up, assessments of the first visual fields were often impaired due to a lack of reliability. Katz et al. (27) found that 19% of normal, 28% of ocular hypertensives, and 37% of glaucoma patients were unreliable on their first visual field. Sherafat et al. (26), in a randomized and controlled trial found that the use of brief video information about the visual field test improved the reliability of the results for those being tested for the first time. In our study this may explain the trend for improvement in visual field abnormalities at 6 months.

Considering visual evoked potentials, the impairment was statistically significant for both fibers explored at 30’ and 60’ angles with a p-value of 0.007 and <0.001 respectively.

Visual evoked potential is an important visual electrophysiological diagnostic exam, which can be used as an objective measure of optic nerve function. Correlations between the magnitude of VEP latency parameters and automated visual parameters have suggested that cortex responses in glaucoma patients could be tested by electrophysiological methods (28). Electrophysiology in glaucoma brings valued information, which detects macular ganglion cell dysfunction and VEP can be of aid in the evaluation of “glaucoma suspects” even before a detectable loss appears by visual field examination (29). Visual evoked potential seems to be the more sensitive exam for detection of visual toxicity. It can be also an interesting exam to describe partial radiation’s effects because it explores different optic fibers.

In our study, D50% was a significant factor in the alteration of 60’VEP. In most of the studies, the dose constraints mainly concerned Dmax or D2% for the optical pathways, considering as a whole these organs as so-called “serial” organs. In term of Dmax delivered on optic pathways we were below the dose constraints usually described since in our study the median prescription dose to the optic pathways was 54Gy (RBE). Usually the dose constraint for Dmax is between 54 et 60Gy (EQD2). But in the study published by Ozkaya et al^4^, visual field and contrast sensitivity were affected significantly with a volume receiving more than 55Gy (V55) >50% of the OAR volume, and a Dmean > 50Gy. Visual evoked potential latency was affected significantly with Dmean > 50Gy, D5% > 55Gy, and Dmax > 60Gy. These results are consistent with the VEP toxicities data obtained in our study.

In our study, we noted no significant change in OCT results after 6 months of treatment. As report in literature the damage to the optic nerve observed by the reduction of the ganglion cell layer and the thickness of retinal fiber revealed by OCT correlates with the VEP latency parameters (28). However, these anomalies are later and appear after the impairment of the visual field. Optical coherence tomography would be more useful for the characterization of the RIONs found in our patients but does not seem to be an appropriate screening test for early toxicities.

The most surprising result observed is the one concerning the link between optical structures distance and toxicities on VEPs. Indeed, in patients with toxicity, the distance of the target volume from the optical pathways was increased. These results confirm uncertainties concerning RBE of proton therapy, LET appears to be more inhomogeneous, especially in areas close to the target volumes.

## Conclusion

Because of the low demography of proton therapy center, the follow-up of patients after proton therapy is not an easy process to set up but it is necessary to improve our knowledges about the biological effects of proton therapy in real life. Our study which will continue and expand during the coming years, suggests that follow-up with in-depth examinations such as VEP could improve the detection of early abnormalities as a biomarker. This could allow us to consider, in the future, early treatments before irreversible consequences appear. Long-term follow-up is thus necessary to clarify these toxicities. Collaboration between ophthalmologist and radiation oncologist is essential to better understand the characteristics of optic neuropathies and to pursue research with more specific and standardized examinations.

## Data Availability Statement

The raw data supporting the conclusions of this article will be made available by the authors, without undue reservation.

## Ethics Statement

The studies involving human participants were reviewed and approved by CNIL. The patients/participants provided their written informed consent to participate in this study.

## Author Contributions

ML: first author, bibliography, data collection, interpretation of ophthalmological examinations, writing of the manuscript. PL: physician, participation of follow-up, help for redaction and submission. JS: statistics and methodology. JBa: physician, methodology, help for redaction and submission. DS: physician, participation of follow-up. WK: physician, participation of follow-up. TP: data collection. AV: contribution in physic and technic interpretation and collection data. PD: collection dosimetric data. JBo: data collection. PM: performed ophthalmologic examination and follow-up. JT: original idea of the project, coordination, methodologist. JQ: performed ophthalmologic examination and follow-up, interpretation of ophthalmological results, coordination, methodologist. All authors contributed to the article and approved the submitted version.

## Conflict of Interest

The authors declare that the research was conducted in the absence of any commercial or financial relationships that could be construed as a potential conflict of interest.
